# Robot-assisted laparoscopic retroperitoneal lymph node dissection with concomitant inferior vena cava thrombectomy for metastatic mixed testicular germ cell cancer: a case report

**DOI:** 10.1186/s13256-019-2200-y

**Published:** 2019-08-27

**Authors:** Kai Zhang, Gang Zhu, Xingshuai Liu, Jiangke Tian, Yanfei Gu, Mengyao Zhai, Lu Yang, Wei Liu, Hongbo Li, Francisco J. Martinez Portillo

**Affiliations:** 1 Department of Urology, Beijing United Family Hospital and Clinics, Beijing, 100015 China; 2 Department of Anesthesiology, Beijing United Family Hospital and Clinics, Beijing, 100015 China; 3 Department of Radiology, Beijing United Family Hospital and Clinics, Beijing, 100015 China; 4 Department of Oncology, Beijing United Family Hospital and Clinics, Beijing, 100015 China; 5 Department of Vascular Surgery, Beijing United Family Hospital and Clinics, Beijing, 100015 China

**Keywords:** Testis cancer, Robotics, Inferior vena cava thrombectomy

## Abstract

**Background:**

The robot-assisted laparoscopic management of post-chemotherapy retroperitoneal metastasis and inferior vena cava tumor thrombus secondary to testicular cancer is a challenging task for urologists.

**Case presentation:**

A pathological examination of a 36-year-old Caucasian man who had undergone a right radical orchiectomy showed mixed testicular germ cell cancer (70% embryonal cancer and 30% seminoma); he had undergone four prior courses of cisplatin, etoposide, and bleomycin chemotherapy and was found to have residual retroperitoneal enlarged lymph nodes close to the right renal hilum and a 9.8 cm inferior vena cava tumor thrombus (pT1, N2, M1, S2). Pre-surgical three-dimensional image reconstruction was performed based on contrast computed tomography data. The inferior vena cava tumor thrombus was found in the vena cava at the level of the celiac trunk and the inferior mesenteric artery.

Our patient accepted treatment with robot-assisted laparoscopic retroperitoneal lymph node dissection with concomitant inferior vena cava thrombectomy and cava reconstruction on September 12, 2018. During the procedure, a drop-in robotic ultrasound probe was used to define the thrombus. Vena cavoscopy using a flexible ureteroscope found that the tumor thrombus adhered to the cava wall in all directions. The tumor thrombus was dissected free from the inferior vena cava lumen, and vena cava reconstruction was achieved using the da Vinci™ Si HD surgical system.

The operative time was 550 minutes. The intraoperative estimated blood loss was 2300 ml. Intraoperative blood transfusions consisted of 10 units of red blood cells (Clavien-Dindo grade II). No Clavien-Dindo grade III or above perioperative complications occurred. The length of hospital stay was 7 days. Pathology revealed no viable cancer cells in any of the residual lymph node tissues or in the vena cava tumor thrombus.

**Conclusion:**

This is the first case of robot-assisted laparoscopic retroperitoneal lymph node dissection with concomitant inferior vena cava thrombectomy and reconstruction for metastatic mixed testicular germ cell cancer published to date. This complicated surgical procedure was facilitated by the innovative usage of three-dimensional image reconstruction for defining the vena cava tumor thrombus, a robotic ultrasound probe for intraoperatively defining the vena cava tumor thrombus, and vena cavoscopy using a flexible ureteroscope.

## Background

Testicular germ cell tumors are among the most curable solid cancers in terms of combined therapy [1]. In some cases, metastatic progression into the retroperitoneal area or, more rarely, a tumor thrombus in the vena cava, has been reported in the literature. It is very common to find a retroperitoneal residual mass of germ cell tumors after chemotherapy. Retroperitoneal lymph node dissection (RPLND) is recommended for nonseminomatous germ cell tumors (NSGCTs) with a residual mass of over 1 cm after chemotherapy on radiographic imaging according to the European Association of Urology (EAU) guidelines. Previous studies of laparoscopic RPLND (L-RPLND) have shown feasibility but difficulty because of perinodal and perivessel fibrosis and adhesions [2, 3]. Robot-assisted L-RPLND (R-RPLND) has proven its advantages with regard to high-definition three-dimensional visualization, increased freedom of instrument movement, and minimization of tremors. It also provides more comfort and dexterity to the surgeon. It overcomes the technical limitations of conventional laparoscopy and can achieve superior oncologic results and surgical safety. The feasibility and effectiveness of robotic techniques in testicular cancer residual mass management have already been assessed [4–7]. However, there have been no studies on R-RPLND with concomitant inferior vena cava (IVC) cancer thrombus thrombectomy for testicular cancer. Here we present our experience with R-RPLND and concomitant IVC thrombectomy in one case of a post-chemotherapy residual mass of metastatic mixed testicular germ cell cancer applied using a lateral and single-docking approach with the da Vinci™ Si HD surgical system.

## Case presentation

### Clinical history

A 36-year-old Caucasian man underwent a right radical orchiectomy in another hospital in November 2017. A pathological examination showed mixed embryonal carcinoma with 30% seminoma and 70% embryonal cancer. The tumor had invaded the tunica albuginea focally but not the tunica vaginalis, with no involvement of the epididymitis or spermatic cord. The tumor was 1.5 × 1.3 × 1.2 cm in size. The levels of tumor markers, such as human chorionic gonadotropin (HCG), alpha-fetoprotein (AFP), and lactate dehydrogenase (LDH), were in the normal range before and after surgery. In April 2018, he came to our clinic due to upper-right abdominal pain. He was free from other medical illnesses, and he had no family history of cancer. He was an English teacher and had never smoked tobacco or drank alcohol.

### Physical examination

He had normal blood pressure, pulse, and temperature. Laboratory tests showed normal white blood cell count, red blood cell (RBC) count, alanine aminotransferase (ALT), and creatinine levels. His LDH, HCG, and AFP levels were 452 U/L, 0.1 U/L, and 2.46 μg/L, respectively. A computed tomography (CT) scan with contrast in April 2018 showed metastatic retroperitoneal lymphadenopathy near his IVC and a tumor thrombus invading the IVC lumen (1.4 × 1.1 × 13 cm), as well as lung and liver metastasis nodules.

### Diagnosis

The diagnosis was mixed testicular germ cell cancer with an IVC tumor thrombus and lung, liver, and retroperitoneal lymph node metastasis.

### Intervention

He underwent four cycles of cisplatin, etoposide, and bleomycin (BEP; cisplatin 35 mg × 5 days, etoposide 160 mg × 5 days, bleomycin 30 mg × 3 days) chemotherapy starting from May 2018, which resulted in a clinically complete response and disappearance of the lung and liver metastasis nodules. However, according to CT and magnetic resonance imaging (MRI) scans obtained on September 10, 2018, he still had contradictory residual retroperitoneal enlarged lymph nodes 1.8 cm in size close to his IVC at the level of the right renal hilum and a 9.8 cm IVC tumor thrombus (level IIIA), according to the staging system for renal cell carcinoma (RCC) with an IVC tumor thrombus [8, 9].

**Fig. 1:**
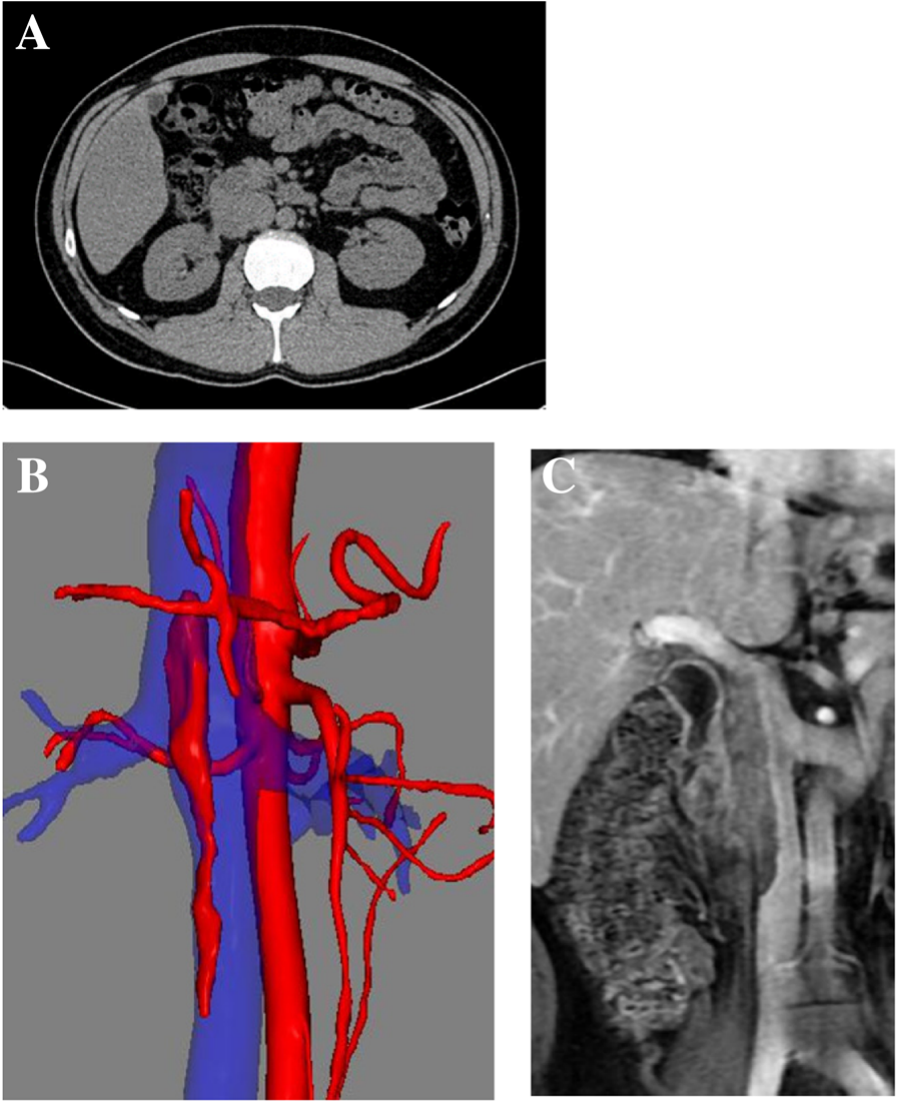
The lymph node enlargement on computed tomography image (**a**) and three-dimensional image reconstruction (**b**) of tumor thrombus in the vena cava and relevant magnetic resonance image (**c**)

Pre-surgical three-dimensional image reconstruction was performed based on contrast CT data. The IVC tumor thrombus was found in his vena cava at the level of the celiac trunk and the inferior mesenteric artery (Fig. 1).

**Fig. 2:**
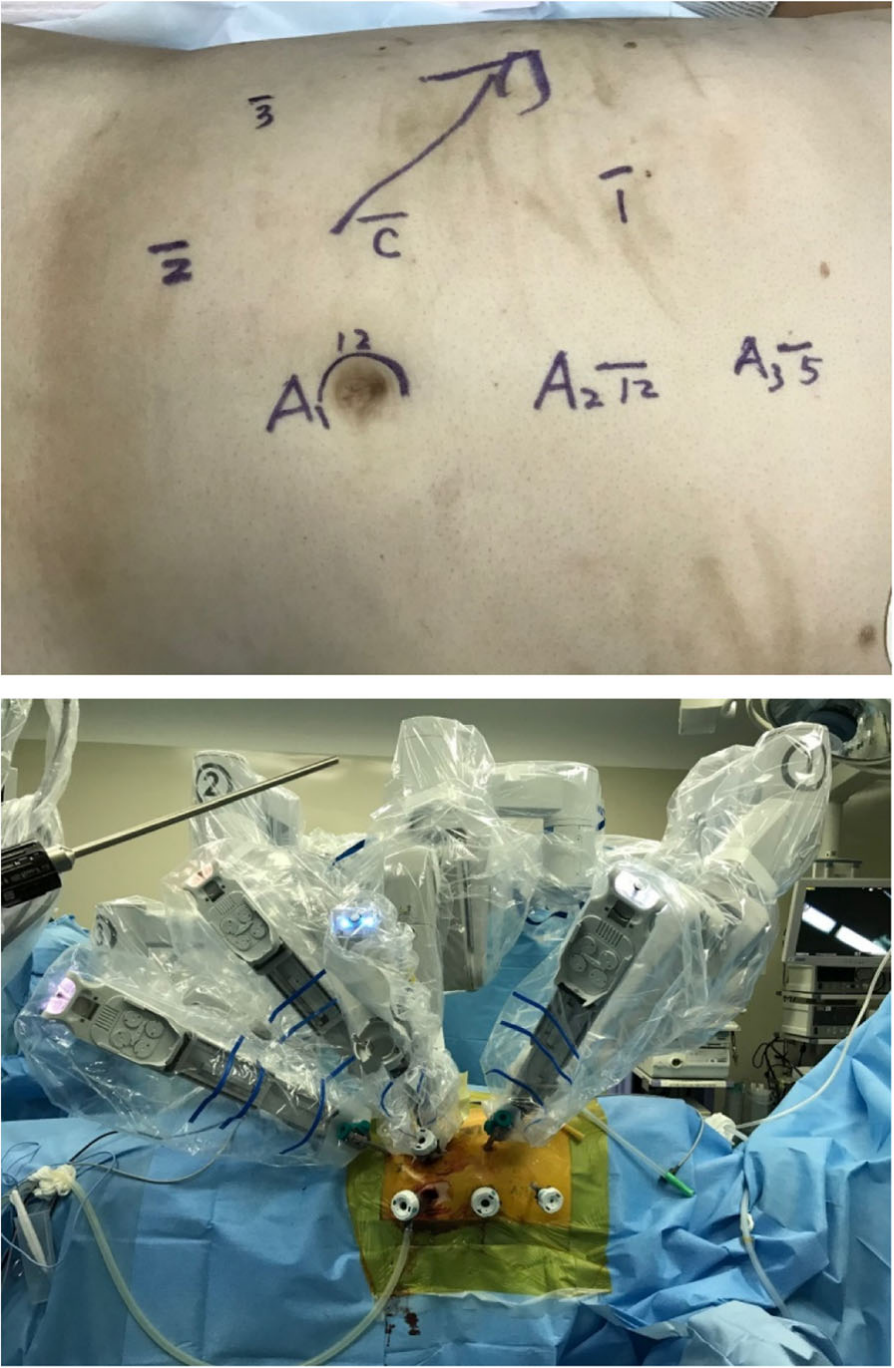
Robotic port placement – a seven-port approach was used, including two assistant ports and one liver retractor port

A four-port right R-RPLND followed by IVC tumor thrombectomy was performed. The robotic port placement and patient position are described in Fig. 2. A seven-port approach was used, including two assistant ports and one liver retractor port. Right unilateral template lymphadenectomy was performed using a nerve-sparing approach, including complete removal of the ipsilateral spermatic cord.

Right R-RPLND was completed in the standard fashion. There were extensive adhesions, and several enlarged lymph nodes around his vena cava and right renal hilum were identified and removed.

IVC tumor thrombectomy and reconstruction were then performed as follows. Dissection of the infrarenal IVC involved the control of all relevant lumbar veins, which were ligated with Hem-o-lok clips and cut. Dissection was carried out in a cephalad direction within the aortocaval region. The IVC at the level of the inferior mesenteric artery was encircled with a double vessel loop tourniquet passing through a half-inch (12.7 mm)-long piece of 20 F rubber tube and secured in place with a Hem-o-lok clip. The left renal vein was mobilized and encircled with a tourniquet. The right renal hilum was dissected, the right renal vein was exposed and encircled with a tourniquet, and the right renal artery was dissected and made ready for bulldog clamp control.

For proximal IVC control, careful aortocaval dissection was performed toward the liver. The right central adrenal vein was controlled with Hem-o-lok clips, and the right lateral border of the suprarenal IVC was dissected. For this level IIIA tumor thrombus, there were no relevant short hepatic veins in the surgical area. A double-fenestrated grasper was used to encircle the IVC with a tourniquet at the retrohepatic location.

**Fig. 3:**
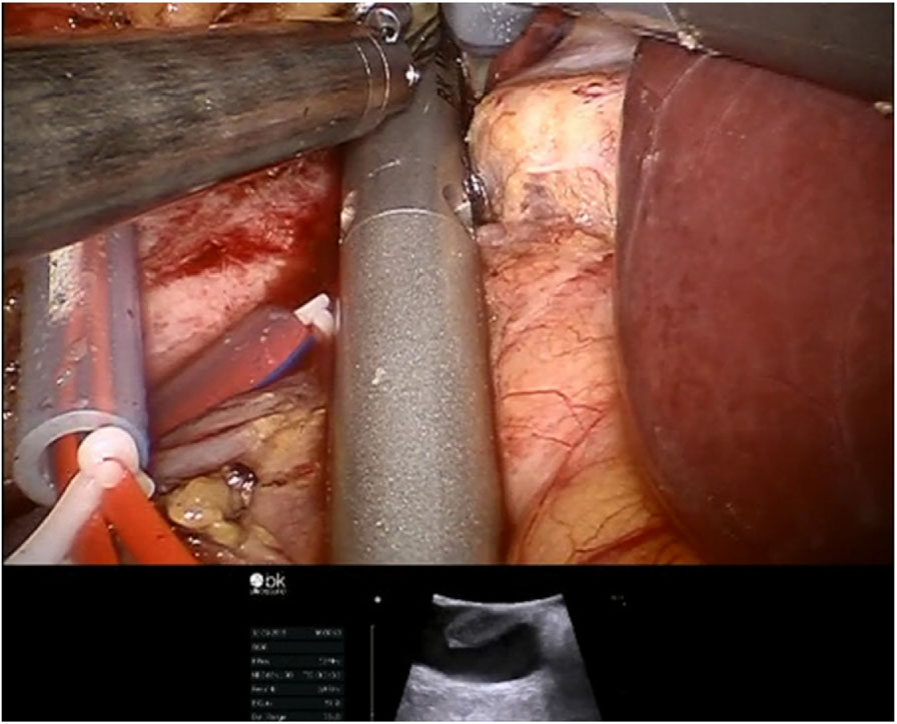
A drop-in robotic ultrasound probe was used for defining the border of vena cava tumor thrombus

All tourniquets were visually reconfirmed to be in the appropriate position with a sufficient margin around the thrombus defined by a drop-in robotic ultrasound probe (ProART™ Robotic Transducer, BK Medical) (Fig. 3). Transesophageal ultrasound was used to monitor the potential for tumor thrombus dislodgement and air emboli.

**Fig. 4:**
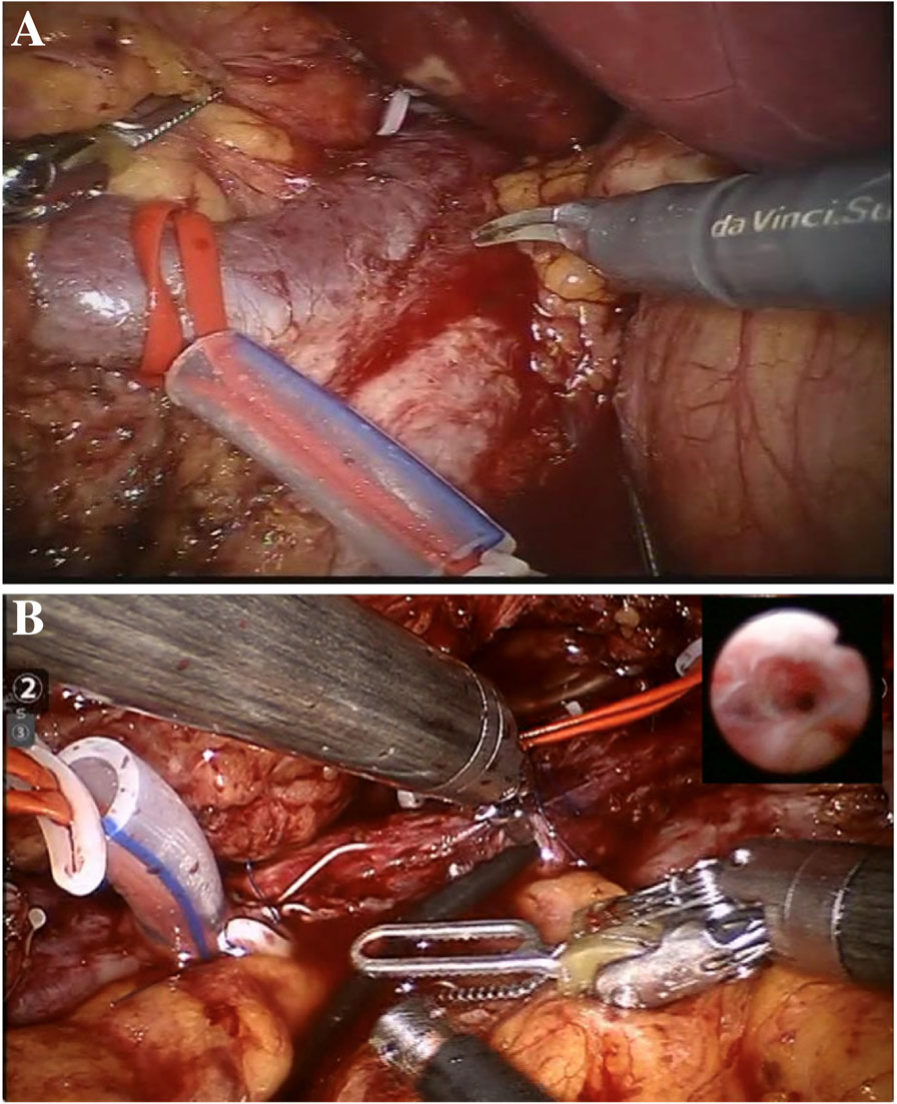
The control of inferior vena cava (**a**) and vena cavoscopy by flexible ureteroscope was used to observe the status and relation of the thrombus with the cava wall (**b**)

Anesthesiologists were alerted that vena cava blood flow would be temporarily halted. We first cinched the distal IVC tourniquet. Once the anesthesiologists assured that our patient was able to tolerate cava clamping and showed no significant hemodynamic changes, the left renal vein, right renal artery, right renal vein, and proximal IVC tourniquets were cinched sequentially to exclude the tumor thrombus-bearing cava segment. The right renal artery was clamped with bulldog clamps. Cavotomy was carried out at the level of the interior mesenteric artery from the assistant port (Fig. 4). Through vena cavoscopy using a flexible ureteroscope (P5, F5.3/8.4, Olympus, Tokyo, Japan), we found that the tumor thrombus adhered to the cava wall in all directions. The cavotomy was extended along the right edge of the IVC past the right renal vein ostium and toward the upper cava tourniquet, for a total length of approximately 10 cm. There was bleeding from the vena cava, and the tourniquets were tightened again; then, there was no more active bleeding.

**Fig. 5:**
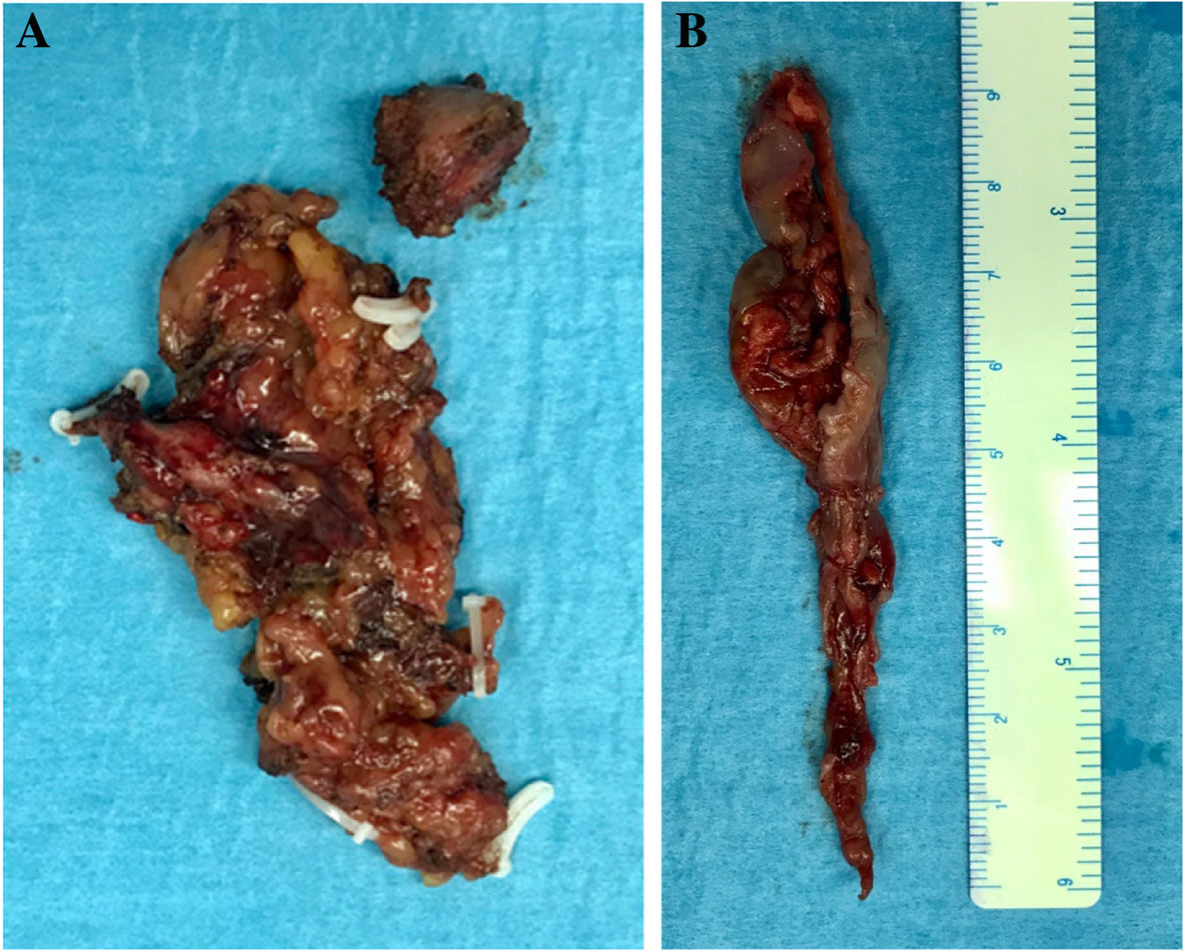
Specimen of retroperitoneal lymph nodes (**a**) and inferior vena cava tumor thrombus (**b**)

The thrombus was reddish and tightly adhered to the vena cava wall. It was carefully dissected free from the IVC wall without local spillage (Fig. 5). The IVC lumen was irrigated with heparinized saline. Cava reconstruction was performed with 5-0 Gore-Tex® sutures (W.L. Gore & Associates, Newark, DE, USA) with a single-layer running stitch. The tourniquets were released sequentially (proximal IVC, left renal vein, right renal vein, right renal artery, distal IVC), and cava flow was restored. The ischemia time for his right kidney was 40 minutes. There was right renal vein bleeding after restoration of the renal artery. The leakage was repaired with 4-0 proline sutures (Johnson & Johnson Medical NV, Belgium). His right kidney was preserved.

After decreasing the abdominal pressure, there was no active bleeding. A 20 F free-drainage tube was placed. The specimens were removed using an Endo Catch™ pouch. The wounds were closed accordingly.

### Outcomes

The operative time was 550 minutes, and the estimated blood loss was 2300 ml with the transfusion of 10 units of RBCs. Postoperatively, he was placed in an intensive care unit (ICU) for 4 days. Subcutaneous heparin was prescribed at a dose of 5000 IU every 8 hours. There were no postoperative complications, and he was discharged on day 7 after the operation. Prophylactic anticoagulation treatment with 40 mg of enoxaparin sodium daily was continued for 1 month. A pathological examination showed no viable cancer cells in the residual tissue, lymph nodes, or vena cava tumor thrombus, which indicated that the treatment for the tumor resulted in a complete response.

Follow-up observations: A routine postoperative clinic visit 2 weeks after discharge revealed no wound infection, stable vital signs, and free mobilization. His erectile function and ejaculation status were normal, without retrograde ejaculation, at 1 month after surgery. CT, chest X-ray, and laboratory investigations at 3 and 6 months showed no abnormalities.

## Discussion and conclusions

We present a case of metastatic testicular cancer treated with R-RPLND and concomitant IVC thrombectomy. To the best of our knowledge, this is the first such case to be reported worldwide.

L-RPLND was first described in 1992 and was initially performed for diagnostic purposes in the post-chemotherapy setting [10]. Since then, L-RPLND has been developed for therapeutic purposes, resulting in similar staging accuracy and oncologic control outcomes as the traditional open technique [11–14]. However, we must face the reality that L-RPLND is technically demanding and requires extensive laparoscopic surgery expertise and an extended learning curve.

R-RPLND has been assessed and proven to offer similar advantages as L-RPLND. These include a shorter hospital stay, quicker return of bowel function, and improved convalescence [15, 16]. R-RPLND has also been approved for selected candidates undergoing neoadjuvant chemotherapy [17–20]. The robotic system allows the surgeon to dissect nodal tissue posterior to or clinging to the vena cava, which could be challenging with conventional laparoscopy.

Our case supports the previous achievements of R-RPLND in conducting meticulous lymph node dissection; there was no more than 50 ml of blood loss at the time we completed the R-RPLND.

As there are no staging systems for a metastatic testicular cancer IVC tumor thrombus available in the literature, we used the Mayo scheme for a RCC IVC tumor thrombus. In this case, the proximal vena cava thrombus was above the first porta hepatis but not the second porta hepatis, and it was staged IIIa [8, 9].

We used three-dimensional image reconstruction in this case, which provided us with direct three-dimensional images of the status of the vessels and thrombus, including the length and border of the thrombus. The three-dimensional reconstruction could be reviewed at any time during surgery, which saved the surgeon from needing to establish unreliable virtual three-dimensional images in his mind based on cross-sectional images. The ultimate goal of three-dimensional image reconstruction is to combine the real-time surgical situation with CT images to facilitate surgical navigation.

We innovatively applied a robotic ultrasound probe to define the superior and inferior borders of the vena cava tumor thrombus. Compared to transesophageal ultrasound, robotic ultrasound can be used more easily to reveal the caudal and cranial cava thrombus borders under direct vision without additional invasiveness. To the best of our knowledge, this is the first report of the use of a robotic ultrasound probe in robotic IVC tumor thrombectomy.

Vena cavoscopy using a flexible ureteroscope was also applied in this case to observe the status and relation of the thrombus to the cava wall. In this case, as the cava lumen was nearly fully obstructed by the tumor thrombus, it was difficult to observe the entire length of the thrombus, but vena cavoscopy did provide some information useful for decision making in extending the cavotomy. It could potentially help in the case of a non-adhered thrombus, and a milking technique could be applied to avoid opening the cava along the length of the thrombus [21].

Robotic IVC thrombectomy was reported previously for the treatment of RCC with IVC tumor thrombus and required a full understanding of applied anatomy and hemodynamics [22–24]. With the accumulation of experience, it is a feasible and safe surgical procedure for highly experienced institutes. Unlike an RCC tumor thrombus, in this mixed testicular germ cell tumor case, the tumor thrombus was longer than 9.8 cm and crossed the bilateral renal veins, nearly blocking the whole IVC. It also adhered to the cava wall, making it impossible to use the milking or balloon retraction technique. The cavotomy in the cava wall extended nearly 10 cm. All of these features made this case challenging because of the risk of significant hemorrhage, thrombus dislodgement, pulmonary embolization, and loss of the right kidney. An artificial vascular patch was prepared in case we could not suture the IVC. However, the defect of the cava wall was not large, so use of the artificial vascular patch was not necessary. There have been previous reports of open series [25]; however, there have been no reports of robotic IVC thrombectomy for testicular cancer vena cava tumor thrombus.

No viable cancer cells were observed in the lymph nodes or thrombus by histopathology, which demonstrated the effectiveness of chemotherapy; however, it also brought into question the necessity of surgery. The positron emission tomography (PET)-CT performed 10 days before the operation showed multiple metastatic lymph nodes in the region of the IVC, and those in the right iliac vessel area had become smaller or disappeared, with normal fluorodeoxyglucose (FDG) uptake. Even the IVC tumor thrombus was no longer visible on the images. However, CT still showed the thrombosis. According to the EAU guidelines, post-chemotherapy surgery is always demanding. Patients undergoing such complex surgeries benefit from disease control despite the greater risk of complications [26, 27].

For this young patient, minimal morbidity, a short convalescence time, and improved quality of life are essential. A short convalescence time may also allow the patient to return to work or receive further chemotherapy earlier if needed.

Further studies with larger cohorts are needed to confirm the safety of this procedure and the long-term oncologic outcomes.

Our study is limited in that it is a retrospective review of only one case. However, this example demonstrates that R-RPLND with concomitant IVC thrombectomy is safe and follows the oncologic principles of the open approach.

R-RPLND with concomitant IVC thrombectomy can be performed successfully and provides improved visualization and dexterity compared with the conventional laparoscopic procedure.

## Data Availability

A Surgery in Motion video accompanying this article can be provided.

## Abbreviations

AFP: Alpha-fetoprotein
ALT: Alanine aminotransferase
BEP: Cisplatin, etoposide, and bleomycin
CT: Computed tomography
EAU: European Association of Urology
FDG: Fluorodeoxyglucose
HCG: Human chorionic gonadotropin
ICU: Intensive care unit
IVC: Inferior vena cava
LDH: Lactate dehydrogenase
L-RPLND: Laparoscopic retroperitoneal lymph node dissection
NSGCT: Nonseminomatous germ cell tumor
PET: Positron emission tomography
RBC: Red blood cell
RCC: Renal cell carcinoma
RPLND: Retroperitoneal lymph node dissection
R-RPLND: Robot-assisted laparoscopic retroperitoneal lymph node dissection

## References

[1] Albers P, Albrecht W, Algaba F, Bokemeyer C, Cohn-Cedermark G, Fizazi K, *et al.* Guidelines on Testicular Cancer: 2015 Update. Eur Urol. 2015;68(6):1054–68. 10.1016/j.eururo.2015.07.044 26297604

[2] Nicolai N, Tarabelloni N, Gasperoni F, Catanzaro M, Stagni S, Torelli T, *et al.* Laparoscopic Retroperitoneal Lymph Node Dissection for Clinical Stage I Nonseminomatous Germ Cell Tumors of the Testis: Safety and Efficacy Analyses at a High Volume Center. J Urol. 2018;199(3):741–7. 10.1016/j.juro.2017.09.088 28964782

[3] Faria EF, Neves HS, Dauster B, Machado RD, Magnabosco WJ, Muller RL, *et al.* Laparoscopic Retroperitoneal Lymph Node Dissection as a Safe Procedure for Postchemotherapy Residual Mass in Testicular Cancer. J Laparoendosc Adv Surg Tech A. 2018;28(2):168–73. 10.1089/lap.2017.0381 29215949

[4] Werntz RP, Pearce SM, Eggener SE. Indications, evolving technique, and early outcomes with robotic retroperitoneal lymph node dissection. Curr Opin Urol. 2018;28(5):461–8. 10.1097/MOU.0000000000000530 29979235

[5] Schwen ZR, Gupta M, Pierorazio PM. A Review of Outcomes and Technique for the Robotic-Assisted Laparoscopic Retroperitoneal Lymph Node Dissection for Testicular Cancer. Adv Urol. 2018;2018:2146080. 10.1155/2018/2146080 PMC5960558 29853869

[6] Pearce SM, Golan S, Gorin MA, Luckenbaugh AN, Williams SB, Ward JF, *et al.* Safety and Early Oncologic Effectiveness of Primary Robotic Retroperitoneal Lymph Node Dissection for Nonseminomatous Germ Cell Testicular Cancer. Eur Urol. 2017;71(3):476–82. 10.1016/j.eururo.2016.05.017 27234998

[7] Stepanian S, Patel M, Porter J. Robot-assisted Laparoscopic Retroperitoneal Lymph Node Dissection for Testicular Cancer: Evolution of the Technique. Eur Urol. 2016;70(4):661–7. 10.1016/j.eururo.2016.03.031 27068395

[8] Blute ML, Leibovich BC, Lohse CM, Cheville JC, Zincke H. The Mayo Clinic experience with surgical management, complications and outcome for patients with renal cell carcinoma and venous tumour thrombus. BJU Int. 2004;94(1):33–41. 10.1111/j.1464-410X.2004.04897.x 15217427

[9] Ciancio G, Vaidya A, Savoie M, Soloway M. Management of renal cell carcinoma with level III thrombus in the inferior vena cava. J Urol. 2002;168(4 Pt 1):1374–7. 10.1016/S0022-5347(05)64452-7 12352396

[10] Rukstalis DB, Chodak GW. Laparoscopic retroperitoneal lymph node dissection in a patient with stage 1 testicular carcinoma. J Urol. 1992;148(6):1907–9. discussion 9-10 10.1016/s0022-5347(17)37068-4 1433638

[11] Busch J, Magheli A, Erber B, Friedersdorff F, Hoffmann I, Kempkensteffen C, *et al.* Laparoscopic and open postchemotherapy retroperitoneal lymph node dissection in patients with advanced testicular cancer--a single center analysis. BMC Urol. 2012;12:15. 10.1186/1471-2490-12-15 PMC3431976 22651395

[12] Abdel-Aziz KF, Anderson JK, Svatek R, Margulis V, Sagalowsky AI, Cadeddu JA. Laparoscopic and open retroperitoneal lymph-node dissection for clinical stage I nonseminomatous germ-cell testis tumors. J Endourol. 2006;20(9):627–31. 10.1089/end.2006.20.627 16999613

[13] Poulakis V, Skriapas K, de Vries R, Dillenburg W, Ferakis N, Witzsch U, *et al.* Quality of life after laparoscopic and open retroperitoneal lymph node dissection in clinical Stage I nonseminomatous germ cell tumor: a comparison study. Urology. 2006;68(1):154–60. 10.1016/j.urology.2006.01.023 16820195

[14] Janetschek G, Hobisch A, Holtl L, Bartsch G. Retroperitoneal lymphadenectomy for clinical stage I nonseminomatous testicular tumor: laparoscopy versus open surgery and impact of learning curve. J Urol. 1996;156(1):89–93. discussion 4 10.1016/s0022-5347(01)65948-2 8648847

[15] Kunit T, Janetschek G. Laparoscopic and robotic postchemotherapy retroperitoneal lymph node dissection. Curr Opin Urol. 2014;24(2):162–7. 10.1097/MOU.0000000000000028 24445557

[16] Harris KT, Gorin MA, Ball MW, Pierorazio PM, Allaf ME. A comparative analysis of robotic vs laparoscopic retroperitoneal lymph node dissection for testicular cancer. BJU Int. 2015;116(6):920–3. 10.1111/bju.13121 25781349

[17] Corona Montes VE, Pastore AL, Gausa L, Rodriguez-Faba O, Breda A, Palou J. Robot assisted retroperitoneal lymph-node dissection after adjuvant therapy: different indications. Minerva Urol Nefrol. 2017;69(2):153–8. 10.23736/S0393-2249.16.02740-5 27681492

[18] Bora GS, Panwar P, Mavuduru RS, Devana SK, Singh SK, Mandal AK. Post chemotherapy retroperitoneal lymph node dissection in germ cell tumor: robotic way. J Robot Surg. 2017;11(2):279–85. 10.1007/s11701-016-0631-7 27503429

[19] Kamel MH, Littlejohn N, Cox M, Eltahawy EA, Davis R. Post-Chemotherapy Robotic Retroperitoneal Lymph Node Dissection: Institutional Experience. J Endourol. 2016;30(5):510–9. 10.1089/end.2015.0673 26669219

[20] Sharma P, Sverrisson EF, Zargar-Shoshtari K, Fishman MN, Sexton WJ, Dickinson SI, *et al.* Minimally invasive post-chemotherapy retroperitoneal lymph node dissection for nonseminoma. Can J Urol. 2015;22(4):7882–9. 26267026

[21] Kundavaram C, Abreu AL, Chopra S, Simone G, Sotelo R, Aron M, *et al.* Advances in Robotic Vena Cava Tumor Thrombectomy: Intracaval Balloon Occlusion, Patch Grafting, and Vena Cavoscopy. Eur Urol. 2016;70(5):884–90. 10.1016/j.eururo.2016.06.024 27423505

[22] Ball MW, Gorin MA, Jayram G, Pierorazio PM, Allaf ME. Robot-assisted radical nephrectomy with inferior vena cava tumor thrombectomy: technique and initial outcomes. Can J Urol. 2015;22(1):7666–70. 25694017

[23] Wang B, Li H, Ma X, Zhang X, Gu L, Li X, *et al.* Robot-assisted Laparoscopic Inferior Vena Cava Thrombectomy: Different Sides Require Different Techniques. Eur Urol. 2016;69(6):1112–9. 10.1016/j.eururo.2015.12.001 26706105

[24] Abaza R. Initial series of robotic radical nephrectomy with vena caval tumor thrombectomy. Eur Urol. 2011;59(4):652–6. 10.1016/j.eururo.2010.08.038 20846783

[25] Hakky T, Kim T, Rodriguez AR, Armstrong P, Mangar D, Spiess PE. Retroperitoneal lymph node dissection with concomitant IVC thrombectomy, caval wall resection, and grafting for metastatic NSGCT. Int Braz J Urol. 2012;38(1):135. discussion 6 10.1590/s1677-55382012000100020 22397783

[26] Ehrlich Y, Kedar D, Zelikovski A, Konichezky M, Baniel J. Vena caval reconstruction during postchemotherapy retroperitoneal lymph node dissection for metastatic germ cell tumor. Urology. 2009;73(2):442.e17–9. 10.1016/j.urology.2008.02.054 18436290

[27] Heidenreich A, Haidl F, Paffenholz P, Pape C, Neumann U, Pfister D. Surgical management of complex residual masses following systemic chemotherapy for metastatic testicular germ cell tumours. Ann Oncol. 2017;28(2):362–7. 10.1093/annonc/mdw605 27831507

